# Resveratrol Analogs Ameliorate Mitochondrial Impairment and Insulin Resistance in a Streptozotocin-Induced In Vitro Model of Alzheimer’s Disease

**DOI:** 10.3390/ijms262110787

**Published:** 2025-11-06

**Authors:** Alexandra Paszternák, Kamilla Varga, Ramá Gyöngyössy, Katinka Tarnóczi, Noémi Sikur, Éva Szökő, Tamás Tábi

**Affiliations:** 1 Department of Pharmacodynamics, Semmelweis University, 4 Nagyvárad tér, H-1089 Budapest, Hungary; paszternak.alexandra@semmelweis.hu (A.P.); tarnoczi.katinka.timea@semmelweis.hu (K.T.); szoko.eva@semmelweis.hu (É.S.); 2 Center for Pharmacology and Drug Research & Development, Semmelweis University, 26 Üllői út, H-1085 Budapest, Hungary

**Keywords:** Alzheimer’s disease, resveratrol, oxyresveratrol, monomethyl resveratrol, trimethyl resveratrol, streptozotocin

## Abstract

Alzheimer’s disease (AD) is characterized by mitochondrial dysfunction, oxidative stress, insulin resistance, and aberrant protein aggregation. Neurodegeneration model with neuronal insulin resistance was induced in SH-SY5Y human neuroblastoma cells by streptozotocin (STZ). We evaluated the neuroprotective effects of resveratrol (RZV) and three structural analogs: oxyresveratrol (OXI), monomethyl resveratrol (MONO), and trimethyl resveratrol (TRI). Mitochondrial function, plasma membrane integrity, oxidative stress) and autophagy were studied by fluorescent assays. Phosphorylated GSK3 levels were measured by ELISA as an indicator of insulin sensitivity. TRI exhibited significant mitochondrial protective effects and strongly induced autophagy. OXI demonstrated excellent antioxidant activity but showed no detectable mitochondrial protective or autophagy-inducing effects. RZV and MONO exhibited moderate antioxidant effects along with strong insulin-sensitizing and autophagy-inducing properties. Insulin sensitivity was most potently restored by RZV (IC_50_ = 54 pM) and MONO (IC_50_ = 50 pM), whereas TRI (IC_50_ = 160 pM) was less potent, and OXI (IC_50_ = 97 pM) showed moderate potency. Our findings suggest that the neuroprotective effects of resveratrol analogs significantly depend on their molecular structure and that they exert their beneficial effects through distinct mechanisms. This research may contribute to the development of novel, multi-target compounds for the treatment of neurodegenerative diseases.

## 1. Introduction

Alzheimer’s disease (AD) is the most common form of neurodegenerative dementia, affecting tens of millions of people worldwide, with a steadily increasing prevalence due to the aging population [1]. Histopathologically, AD is characterized by the presence of extracellular β-amyloid (Aβ) plaques and intraneuronal neurofibrillary tangles composed of hyperphosphorylated tau protein in the cerebral cortex and hippocampus, which are key markers of synaptic and neuronal damage [1,2]. However, the pathomechanism of AD is extremely complex. In recent years, it has become increasingly evident that, beyond the amyloid cascade, several other factors—including metabolic and vascular abnormalities, chronic inflammation, and oxidative damage—play central roles in the pathogenesis of the disease [1,3,4,5]. Consequently, AD is now considered a multifactorial neurodegenerative disorder in which age-related metabolic disturbances are particularly important [2,4,5].

Epidemiological and experimental evidence supports that components of the metabolic syndrome—especially type 2 diabetes mellitus (T2DM) and its associated insulin resistance—significantly increase the risk of developing AD [6,7]. A recent meta-analysis found that individuals with T2DM have an approximately 50% higher risk of AD compared to age-matched healthy controls [5]. Insulin also plays a key role in the brain; central insulin signaling supports neuronal survival, synaptic plasticity, and cognition [6,8,9]. In AD, impairment of insulin signaling was clearly demonstrated with reduced expression of insulin receptors and glucose transporters, enhanced inhibitory phosphorylation of IRS, and decreased activation of downstream pathways such as PI3K/Akt signaling [5,8,10]. Cerebral insulin resistance results in disinhibition of glycogen synthase kinase-3β (GSK-3β) that promotes tau hyperphosphorylation and amyloidogenic processing of the amyloid precursor protein (APP); thus, disrupted insulin signaling is linked to the classical AD pathologies [8,9,10]. Furthermore, chronic hyperglycemia and hyperinsulinemia associated with type 2 diabetes (T2DM) elicit oxidative stress, hinder Aβ degradation, and trigger microglia-mediated inflammation that further exacerbates neurodegeneration [3,4,8,11]. Furthermore, insulin-sensitizing therapies and intranasal insulin have been reported to induce cognitive improvements in multiple experimental AD models and early-phase clinical trials [3].

Oxidative stress and disrupted redox homeostasis are fundamental in AD. Signs of oxidative damage appear early in AD, and their markers (lipid peroxidation end products, oxidized proteins, and nucleic acids) rise in the brain and cerebrospinal fluid; meanwhile, antioxidant defenses are often depleted [3,11]. Oxidative stress and insulin resistance reinforce each other; hyperglycemia and mitochondrial dysfunction augment reactive oxygen species (ROS) generation and impair insulin signaling, while the insulin resistance is accompanied by elevated activity of pro-oxidant kinases (GSK-3, JNK), sustaining a vicious cycle [3,8]. Persistent microglial and astrocytic activation creates a pro-inflammatory milieu that promotes Aβ accumulation and neuronal damage [2,12].

Dysfunction of protein homeostasis, particularly the impaired autophagy, is another major contributor to neurodegeneration. Mitophagy, the selective degradation of mitochondria, is critical for neuronal survival, as accumulation of dysfunctional mitochondria leads to elevated ROS levels and energy deficits [13]. Aging and AD are characterized by impaired autophagy and mitophagy. Autophagosome accumulation and lysosomal dysfunction are evident in AD brains, compromising the clearance of Aβ and hyperphosphorylated tau [7,13]. Autophagy-related gene deficiencies (e.g., beclin-1) promote Aβ accumulation and neurodegeneration in experimental models, while enhancing autophagic flux (e.g., via rapamycin or resveratrol) improves cognitive performance and reduces AD-like pathology [2,7,13]. Thus, autophagy represents a promising therapeutic target in AD by promoting the clearance of toxic aggregates and damaged organelles.

In recent years, several review articles have addressed the neuroprotective effects of natural compounds, particularly polyphenols, in neurodegenerative diseases. A recent review discusses the structural features and aggregation of α-synuclein, as well as the ability of various natural polyphenols—such as epigallocatechin gallate (EGCG), baicalein, or nordihydroguaiaretic acid—to interact with the monomeric and oligomeric forms of the protein, thereby inhibiting fibrillar aggregation and formation of toxic intermediates [14]. Although synucleinopathies (e.g., Parkinson’s disease) are the main focus, these mechanisms are also relevant to AD, as protein aggregation, oxidative stress, and inflammatory processes represent common pathomechanisms across different neurodegenerative disorders. In parallel, another comprehensive review highlights that naturally derived bioactive compounds (flavonoids, alkaloids, terpenoids, etc.) can interfere with the pathogenesis of AD at multiple levels. According to the reviewed literature, these molecules can reduce β-amyloid overproduction, attenuate tau hyperphosphorylation, inhibit neuroinflammation and apoptosis, and improve autophagy and mitochondrial function. In addition, through their antioxidant activity, they can alleviate oxidative stress and the related endoplasmic reticulum (ER) stress, which are key factors in the progression of neurodegeneration [15]. Among these natural compounds, resveratrol (trans-3,5,4′-trihydroxystilbene) deserves particular attention as one of the most promising molecules due to its antioxidant, anti-inflammatory, anti-apoptotic, and cognitive-enhancing properties [16,17,18]. Preclinical studies have demonstrated that resveratrol improves cognitive functions, reduces amyloid and tau pathology, and mitigates inflammation and oxidative stress in AD models [19]. In our recent research, we have demonstrated the protective effect of resveratrol in an in vivo insulin-resistant neurodegeneration model developed in our laboratory [8]. It significantly reversed the insulin resistance induced by streptozotocin (STZ) in SH-SY5Y neuroblastoma cells and improved mitochondrial quality control by improving both mitogenesis and autophagy [20]. Despite its beneficial effects, the clinical use of resveratrol is hindered by its poor bioavailability due to its rapid metabolism and limited central nervous system penetration [1,21]. To overcome these limitations, several structural derivatives of resveratrol have been developed. Modifications such as hydroxyl group methylation or the addition of extra hydroxyl groups significantly affect both its pharmacokinetics and pharmacodynamics [22,23]. However, the activity of resveratrol analogs can also differ considerably. According to our previous results, monomethylated resveratrol retains cytoprotective effects, potentially through autophagy induction, despite its reduced antioxidant activity. In contrast, trimethylated resveratrol, which lacks free hydroxyl groups, exhibits no antioxidant effects but even acts as a pro-oxidant. Conversely, the hydroxylated analog oxyresveratrol exhibits stronger antioxidant properties without autophagy induction [23]. Yet, the effect of structural modifications on the neuroprotective activity of resveratrol in the insulin resistance-associated neurodegenerative processes remains unclear, as few studies have evaluated the efficacy of resveratrol analogs in AD-relevant in vitro models [24,25].

In our study, we aimed to compare the neuroprotective effects of three resveratrol analogs—oxyresveratrol, monomethylated resveratrol, and trimethylated resveratrol— to that of the parent compound in an in vitro model of AD based on STZ-induced insulin resistance in retinoic acid-differentiated SH-SY5Y cells [8]. Their ability to counteract key pathological processes of AD—oxidative stress, autophagy dysfunction, and insulin signaling impairment—was studied. Our results are expected to contribute to a better understanding of the structure-activity relationships of resveratrol derivatives and pave the way to the development of multitarget neuroprotective strategies against AD.

## 2. Results

### 2.1. Effect of Resveratrol Derivatives on Oxidative Stress

Throughout the experiments, 1 mM STZ was applied to induce insulin-resistant neurodegeneration. As a control condition (C), culture medium supplemented with 5% FBS was used, which is the same medium applied during differentiation and used also as a vehicle for STZ treatment, according to our previously established protocol [8]. A 10 µM concentration of resveratrol analogs was selected based on our previous results with resveratrol [20].

Changes in ROS generation serve as an important indicator of cellular stress. Therefore, SH-SY5Y cells were exposed to STZ in the presence of resveratrol analogs for 6 and 72 h, and both hydrogen peroxide and superoxide levels were assayed by using their fluorescent indicators.

**Figure 1:**
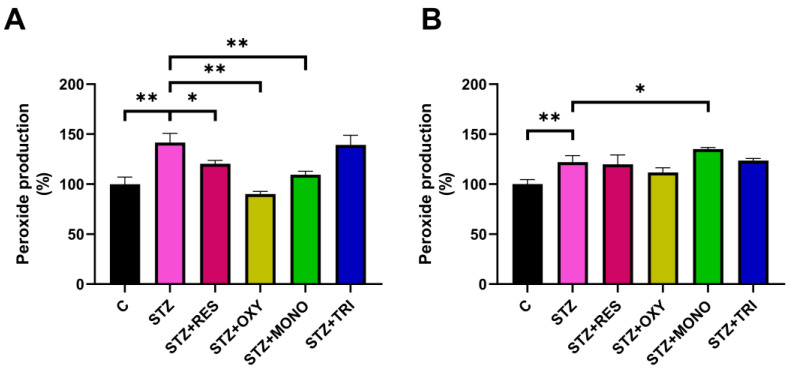
Peroxide production in SH-SY5Y cells after 6 h (**A**) and 72 h (**B**) of treatment with streptozotocin (1 mM) and resveratrol or its analogs (10 µM), measured using 2′,7′-dichlorofluorescin diacetate (DCFDA) fluorescence. Data are expressed as the percentage of the control group. C: control; STZ: streptozotocin; RES: resveratrol; OXY: oxyresveratrol; MONO: monomethyl resveratrol; TRI: trimethyl resveratrol; * *p* < 0.05; ** *p* < 0.001.

At 6 h, STZ treatment resulted in an increase in hydrogen peroxide levels compared to the control. In the presence of resveratrol, a moderate but significant reduction was observed, while oxyresveratrol and monomethyl resveratrol more effectively attenuated the STZ-induced elevation of peroxide levels. Trimethyl resveratrol did not produce any detectable effect at this time point (Figure 1A). By 72 h, the STZ-induced elevation in peroxide production was not significantly reduced by any of the examined compounds; furthermore, it was significantly increased by monomethyl resveratrol treatment (Figure 1B).

**Figure 2:**
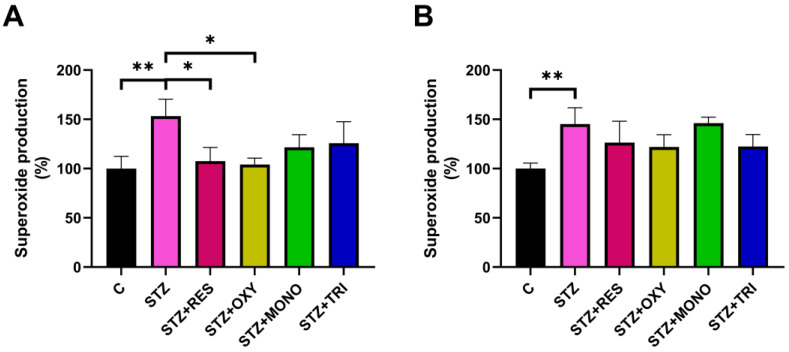
Superoxide production in SH-SY5Y cells after 6 h (**A**) and 72 h (**B**) of treatment with streptozotocin (1 mM) and resveratrol or its analogs (10 µM), measured by dihydroethidium (HE) fluorescence. Data are expressed as the percentage of the control group. C: control; STZ: streptozotocin; RES: resveratrol; OXY: oxyresveratrol; MONO: monomethyl resveratrol; TRI: trimethyl resveratrol; * *p* < 0.05, ** *p* < 0.001.

Similarly to peroxide, STZ treatment led to an increase in superoxide levels at 6 h. Resveratrol and oxyresveratrol mitigated this elevation, whereas monomethyl and trimethyl resveratrol showed no significant effect (Figure 2A). At 72 h, superoxide level was still elevated in the STZ group with no significant effect of any resveratrol analog treatments (Figure 2B).

### 2.2. Effect of Resveratrol Derivatives on Mitochondrial Function

Mitochondrial function was evaluated following STZ treatment in the presence of resveratrol analogs. Mitochondrial membrane potential is a sensitive marker of both early and late cellular stress responses; therefore, SH-SY5Y cells were treated for 6 or 72 h, and JC-1 fluorescence ratios were measured as an estimate. Mitochondrial mass provides further insight into the effects of prolonged stress and was assessed by MitoTracker Green staining following 72 h of treatment.

**Figure 3:**
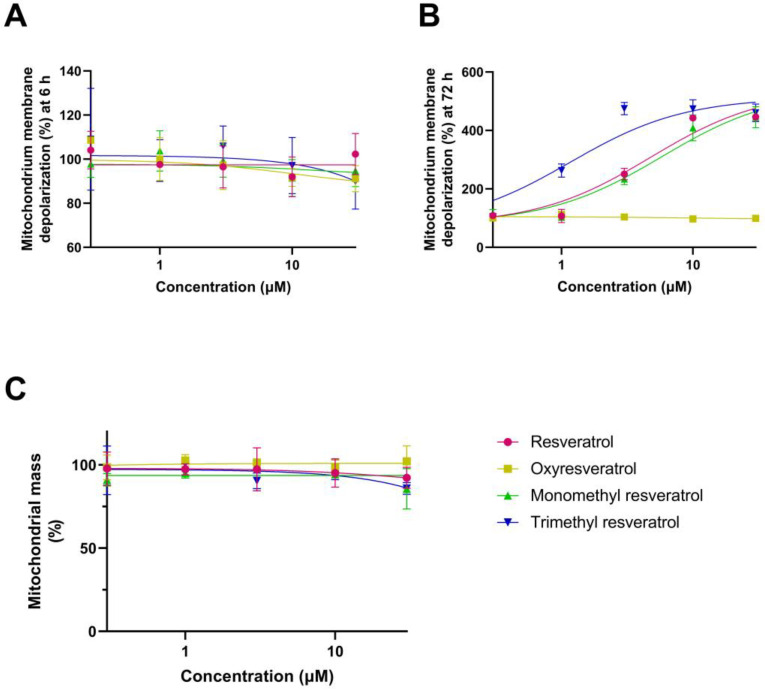
SH-SY5Y cells were treated with streptozotocin (1 mM) and resveratrol or its analogs (0–30 µM), after which mitochondrial membrane potential was estimated by JC-1 fluorescence at 6 h (**A**) and 72 h (**B**), and mitochondrial mass (**C**) was assessed at 72 h using MitoTracker Green fluorescence. Data are expressed as the percentage of the control group.

At 6 h, none of the tested compounds induced detectable changes in mitochondrial membrane potential (Figure 3A). At 72 h, all derivatives except oxyresveratrol induced a concentration-dependent depolarization. Trimethyl resveratrol showed a higher potency and induced depolarization at lower concentrations, while its maximal effect aligned with those of resveratrol and monomethyl resveratrol. Oxyresveratrol did not cause any deviation from baseline at any concentration (Figure 3B).

No changes in mitochondrial mass were observed for any of the tested compounds (Figure 3C).

### 2.3. Effect of Resveratrol Derivatives on Autophagy

To investigate whether STZ treatment and resveratrol analogs influence autophagic activity, SH-SY5Y cells were analyzed by acridine orange staining, quantified by red–green fluorescence ratio.

**Figure 4:**
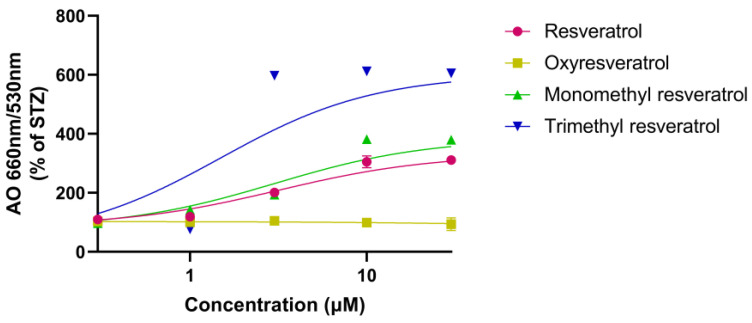
Autophagic vacuole accumulation in SH-SY5Y cells after 72 h of treatment with streptozotocin (1 mM) and resveratrol or its analogs (0–30 µM), measured by acridine orange staining. Fluorescence signals were normalized to cell number, the red/green (660/530 nm) ratio was calculated, and results were expressed as the percentage of the STZ group.

Resveratrol and monomethyl resveratrol induced a similar, concentration-dependent increase in the red/green fluorescence ratio, indicating accumulation of acidic vacuoles. Trimethyl resveratrol was found to induce autophagy more potently and effectively, as evidenced by a higher red–green fluorescence ratio. Oxyresveratrol did not induce any detectable changes across the examined range (Figure 4).

### 2.4. Effect of Resveratrol and Its Derivatives on Protein Aggregation

Protein aggregation, including the accumulation of amyloid-like structures, is a central pathological hallmark of AD. To examine whether STZ treatment and resveratrol analogs influence protein aggregation, SH-SY5Y cells were stained with Thioflavin S, and their fluorescence signals were quantified.

**Figure 5:**
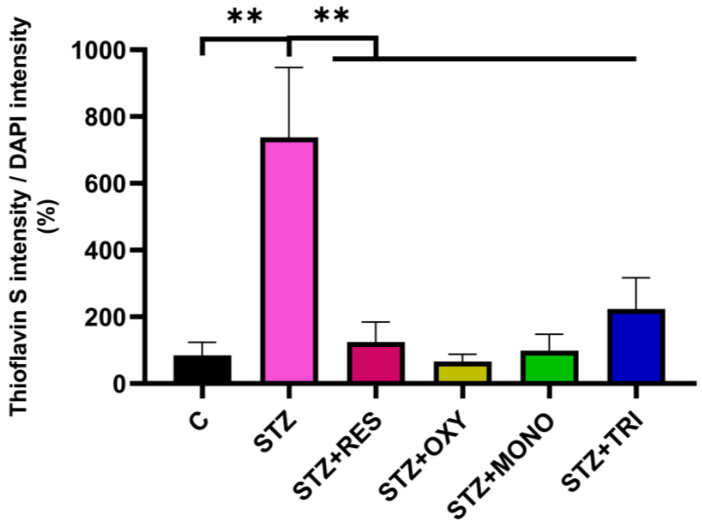
Thioflavin S staining of protein aggregates in SH-SY5Y cells after 72 h of treatment with streptozotocin (1 mM) and resveratrol or its analogs at a concentration of 10 µM. The level of protein aggregates was quantified as the ratio of thioflavin S to DAPI fluorescence intensity. C: control; STZ: streptozotocin; RES: resveratrol; OXY: oxyresveratrol; MONO: monomethyl resveratrol; TRI: trimethyl resveratrol. ** *p* < 0.001.

STZ treatment induced a pronounced increase in fluorescence compared to the control. Co-treatment with resveratrol, monomethyl resveratrol, oxyresveratrol, and trimethyl resveratrol markedly reduced the quantity of protein aggregates to levels similar to those in the control (Figure 5).

### 2.5. Effect of Resveratrol Derivatives on the Cytoprotective Effect of Insulin

To evaluate the involvement of improved insulin resistance in the beneficial effect of resveratrol analogs, the concentration–response relationship of the cytoprotective effect of insulin in their presence was studied. Lactate dehydrogenase (LDH) release was used to assay cell death.

**Figure 6:**
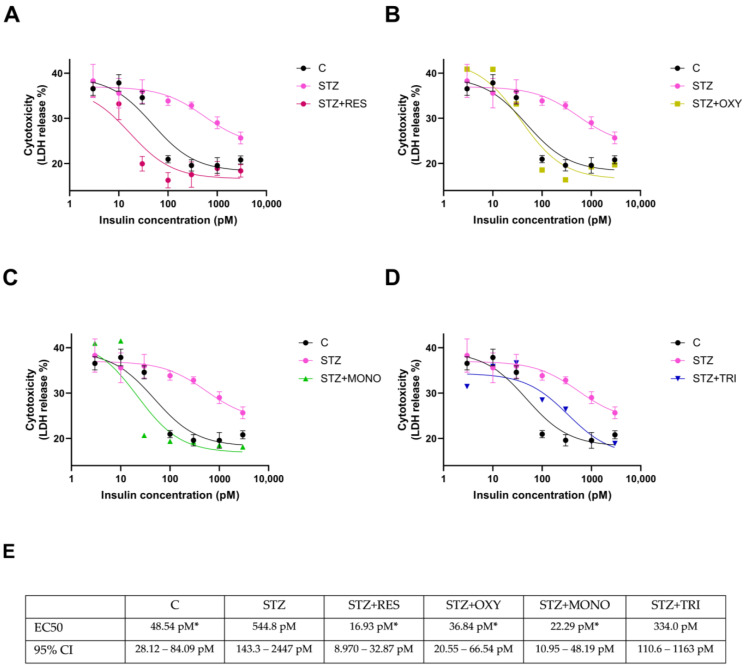
Effects of resveratrol (**A**), oxyresveratrol (**B**), monomethyl resveratrol (**C**), and trimethyl resveratrol (**D**) on LDH release in SH-SY5Y cells after 72 h of treatment. Cells were treated with streptozotocin (1 mM) and resveratrol or its analogs (10 µM), and insulin (0–3000 pM). LDH release was measured as a marker of cytotoxicity and expressed as the percentage of total cell lysis. Data are presented as % cytotoxicity. The curves show the effect of insulin (0–3000 pM) on cytotoxicity. Panel (**E**) shows the EC50 values of the concentration-response curves. C: control; STZ: streptozotocin; RES: resveratrol; OXY: oxyresveratrol; MONO: monomethyl resveratrol; TRI: trimethyl resveratrol. * *p* < 0.05 vs. STZ group.

In accordance with our previous results [8], in the STZ group, although insulin induced a concentration-dependent decrease in LDH release, its neuroprotective potency was significantly decreased compared to that seen in the control, indicating the development of insulin resistance. (Figure 6). In the presence of resveratrol, oxyresveratrol, and monomethyl resveratrol, the insulin responsiveness of the cells was completely restored (Figure 6A–C). Trimethyl resveratrol also improved insulin sensitivity, but it could not completely reverse the effect of STZ on the cytoprotective effect of insulin (Figure 6D).

### 2.6. Effect of Resveratrol Derivatives on Insulin Signaling

To further confirm the effect of resveratrol analogs on insulin signaling, insulin-induced GSK-3β phosphorylation was determined in their presence.

**Figure 7:**
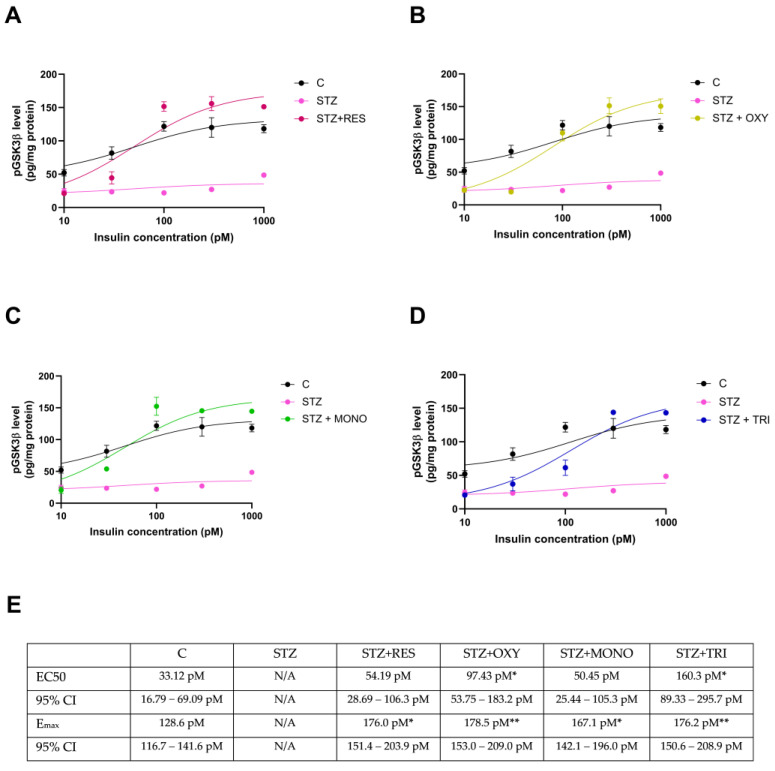
Effects of resveratrol (**A**), oxyresveratrol (**B**), monomethyl resveratrol (**C**), and trimethyl resveratrol (**D**) on GSK3β phosphorylation in SH-SY5Y cells after 72 h of treatment. Panel (**E**) shows the EC50 values of the concentration-response curves. Cells were treated with streptozotocin (1 mM) and resveratrol or its analogs (10 μM), and insulin (0–1000 pM), and phosphorylation of GSK3β was determined after 72 h of treatment. C: control; STZ: streptozotocin; RES: resveratrol; OXY: oxyresveratrol; MONO: monomethyl resveratrol; TRI: trimethyl resveratrol. * *p* < 0.05 vs. C group, ** *p* < 0.001 vs. C group.

Compared to the control group, the concentration-dependent inhibitory phosphorylation of GSK-3β was abolished in the STZ group. Resveratrol and all of its analogs restored insulin responsiveness of the STZ-treated cells. Furthermore, they even increased the efficacy of insulin above the value seen in the case of the control. Trimethyl resveratrol was also somewhat less potent in this experiment than the other compounds but was still able to completely reverse the effect of STZ (Figure 7).

## 3. Discussion

The neurotoxic effect of streptozotocin (STZ) is well documented. It impairs insulin signaling in the nervous system and induces neurodegenerative alterations resembling AD [26]. In our results, STZ induced a significant increase in ROS generation, mitochondrial depolarization, insulin resistance, and accumulation of protein aggregates in line with the previous literature [26,27]. The found alterations are consistent with the AD pathomechanisms and support the applicability of the model for studying the neuroprotective effect of resveratrol analogs.

Resveratrol possesses numerous neuroprotective properties, which have been demonstrated in various neurodegeneration models (for reviews, see [28,29,30,31]). In our previous work, we showed a strong protective effect in the same model. It had effectively reversed insulin resistance induced by STZ treatment via augmenting insulin signaling. It also significantly reduced the levels of ROS, upregulated the antioxidant machineries, and improved the mitochondrial quality control via enhanced autophagy and mitogenesis [20]. In the present study, similarly to resveratrol, its three analogs also showed neuroprotective activity by improving insulin sensitivity and reversing STZ-induced AD-like pathologies, although with different efficacy and potency and likely via different mechanisms. All the examined compounds effectively upregulated the compromised insulin signaling in the STZ model of neurodegeneration. They improved the inhibitory phosphorylation of GSK-3 that is accompanied by a significant reduction in protein aggregates characteristic of neurodegeneration in AD. Phosphorylation of tau protein by GSK-3β and the protective effect of insulin are well established [32,33], while involvement of GSK-3α in amyloid plaque formation is also suggested [34,35]; thus, its inhibition is clearly connected to the anti-AD effect.

Monomethyl resveratrol was the most similar to the parent compound, showing similar antioxidant and autophagy augmenting effects. Both compounds induced a concentration-dependent mitochondrial depolarization that, in turn, may contribute to the induction of autophagy and mitochondrial quality control. The effect of resveratrol on mitochondrial function is extensively discussed in the literature. It has a weak inhibitory effect on ATP synthase [36,37] as well as other mitochondrial electron transport chain complexes [38] was demonstrated. These findings are in line with our previous [20,23,39] and present results. Mild inhibition of the electron transport chain can impair mitochondrial membrane potential and reduce the ATP–AMP ratio. This, in turn, may enhance AMPK activity, which is another proposed mechanism behind the effects of resveratrol [40,41,42,43]. In our previous work, we also demonstrated the importance of AMPK activity in the effect of resveratrol using the same model that may contribute to both improved mitochondrial quality control via upregulating autophagy and reversal of insulin resistance [20]. Activation of AMPK by monomethyl resveratrol was also reported [44] which further confirms the similar mechanism of the two compounds. Induction of autophagy to a similar extent by both resveratrol and its monomethyl analog is found in our present and previous works [23]. Autophagy is involved in multiple cellular processes that can participate in the neuroprotective effect of the examined compounds. It contributes to the elimination of damaged organelles, e.g., mitochondria, and enhances the clearance of pathological protein aggregates (for review, see [45]). A bidirectional connection between insulin resistance and defective autophagy is also proposed [46]; thus, we can speculate the paramount importance of autophagy induction in the insulin-sensitizing and neuroprotective effects of resveratrol and monomethyl resveratrol.

The two compounds also shared some similarity in their antioxidant effect in the early phase of STZ exposure that declined after continued treatment, and in the case of monomethyl resveratrol, even turned to prooxidant activity. As a polyphenolic compound, resveratrol was demonstrated to be able to directly scavenge free radicals [47,48,49], and its effect on endogenous antioxidant machineries, such as superoxide dismutase, glutathione peroxidase, and catalase, was also reported (for review [50]). Activation of Nrf2 signaling was identified as a crucial upstream step in the strengthened antioxidant defense induced by resveratrol [51,52]. Similar free radical scavenger activity [53] and facilitation of the Nrf2 pathway by monomethyl resveratrol [54] were also shown; however, its prooxidant and Nrf2 inhibitory action were also reported [55]. In line with the literature data, our present results suggest that direct scavenger activity of the compounds is more prominent compared to the upregulation of antioxidant enzyme systems because of the short-term effect. Based on the present results, we can infer that although ROS neutralizing activity contributes to the insulin-sensitizing and neuroprotective effect of resveratrol and monomethyl resveratrol, they are more considerably derived from autophagy induction.

Trimethyl resveratrol lacks free phenolic hydroxyl groups, which is consistent with its abolished antioxidant activity and reduced neuroprotective effect found in the present experiments. Direct free radical scavenger activity of stilbene derivatives relies on their phenolic hydroxyl groups, with 4′ one being the most important in the case of resveratrol [56], which can explain the lack of antioxidant activity. This is in line with the report of Kim et al., who demonstrated the failure of trimethyl resveratrol to protect HT22 neuronal cells from glutamate neurotoxicity and associated oxidative stress, contrary to resveratrol [57]. On the other hand, the trimethylated analog is still capable for modulation of mitochondrial membrane potential and inducing autophagy [23], improving AMPK activity [58] and insulin sensitivity [59]. Our results, together with the literature data, suggest that trimethyl resveratrol is a strong activator of autophagy without significant antioxidant activity that can improve insulin resistance and accumulation of protein aggregates; however, its neuroprotective effect lags behind its parent compound.

Oxyresveratrol was found to be a strong antioxidant that can be explained by the presence of an additional phenolic hydroxyl group on the stilbene core [60,61]. Its similar but partially synergistic direct antioxidant effect compared to that of resveratrol was reported [62], indicating some difference in their scavenger mechanism. Enhanced antioxidant defense via activation of Nrf2-mediated transcription was also described in the case of oxyresveratrol [63,64], further confirming its comparable activity to its parent compound. On the other hand, contrary to resveratrol and the other analogs, it had no effect on the mitochondrial membrane potential and was not able to induce autophagy. These results are in line with our previous findings on mouse embryonic fibroblast [23]. Although the induction of autophagy by oxyresveratrol was reported by some papers [25,65], it might be condition-dependent. The considerable effect of the compound on insulin signaling and protein aggregation suggests an alternative neuroprotective mechanism that warrants further studies. Involvement of AMPK activation and alleviation of glutamate toxicity by oxyresveratrol were reported in different neuroprotective models [25,66,67] that might also have contributed to its effect in the present model.

Altogether, our results indicate significant differences in the mechanisms underlying the neuroprotective effects of resveratrol derivatives, summarized in Table 1. Although all the examined compounds reversed insulin resistance induced by STZ treatment, comparing the effects of resveratrol, its methylated and hydroxylated analogs revealed important structure–activity relationship data. The neuroprotective effect of resveratrol is strongly connected to the presence of free hydroxyl groups, which are critical to its antioxidant capacity [68,69]. Its molecular structure, however, is also appropriate for the induction of autophagy [17] and mitochondrial quality control [36,70] likely by influencing the mitochondrial respiratory chain [36,37] and activating AMPK [40,41,42,43]. This dual mechanism—antioxidant defense combined with autophagy induction—explains the complex protective action of the parent compound [23]. The introduction of a single methyl group can reduce the antioxidant capacity of resveratrol by decreasing the number of free hydroxyl groups available for free radical scavenging [53,71]. This structural modification may contribute to its altered redox profile, showing antioxidant activity in the short term [72] but even prooxidant effects at later stages [55]. However, the presence of a single methyl substitution does not modify its autophagy-inducing activity, which explains its substantial similarity to resveratrol [23]. In trimethyl resveratrol, the complete methylation of phenolic hydroxyl groups abolishes its direct antioxidant activity, due to the lack of functional groups required for the free radical scavenging effect [56]. This structural feature accounts for its weaker neuroprotective effect and enhanced ROS generation. On the other hand, it has a more potent effect on the mitochondria and more effectively activates autophagy. The lack of antioxidant capacity, however, still limits its protective potential compared to the parent compound [23]. In the case of oxyresveratrol, the addition of an extra hydroxyl group to the stilbene core enhances the radical scavenging capacity and provides a strong antioxidant activity [60,61,73]. However, this structural modification results in a distinct mechanism of action, as it cannot induce autophagy, and its neuroprotective effect is likely mediated primarily through antioxidant pathways rather than mitochondrial quality control [63].

This study has certain limitations. Protein aggregation was assessed using Thioflavin S staining, which detects β-sheet-rich amyloid-like structures in a protein-independent manner. Therefore, the observed signal was interpreted as a marker of global protein aggregation burden, without assigning it to a specific protein such as tau, Aβ, α-synuclein, or hIAPP. In addition, treatments were administered in parallel with STZ exposure (co-treatment) rather than in a post-treatment setting. While this approach is commonly applied in in vitro studies, it does not fully reproduce therapeutic conditions, where interventions are given after pathological alterations have already developed. Future studies will be required to address these aspects for a more comprehensive evaluation of the neuroprotective potential of resveratrol analogs.

## 4. Materials and Methods

### 4.1. Materials

Resveratrol and its derivatives, including oxyresveratrol, monomethyl resveratrol, and trimethyl resveratrol, were obtained from Tokyo Chemical Industry (Tokyo, Japan). Cell culture medium of DMEM/F12 supplemented with stable glutamine was sourced from VWR International (Radnor, PA, USA), while FBS was purchased from BioSera (Nuaille, France). Trypsin-EDTA, retinoic acid, Triton X-100, dimethyl sulfoxide (DMSO), and various assay reagents were obtained from Merck (Darmstadt, Germany), while STZ was purchased from Cayman Chemical Company (Ann Arbor, MI, USA). Fluorescent dyes and staining reagents, including JC-1, hydroethidine (HE), 2′,7′-dichlorofluorescein diacetate (DCFDA), Mitotracker Green, Hoechst 33342 dye, tioflavin S, paraformaldehyde, and acridine orange (AO) were supplied by ThermoFisher Scientific (Waltham, MA, USA). The phospho-GSK-3 alpha/beta (S21/S9) DuoSet IC ELISA kit was acquired from Bio-Techne (Minneapolis, MN, USA). Insulin was provided by the University Pharmacy of Semmelweis University (Budapest, Hungary).

The tested compounds were dissolved in DMSO, and the final concentration in the cell cultures did not exceed 0.5% in any of the experiments. Control cells were treated with an equal amount of DMSO.

### 4.2. Cell Cultures and Treatments

Human neuroblastoma SH-SY5Y cells (ECACC, UK) were seeded in 96- or 24-well plates, depending on the experiment, and cultured in DMEM/F12 medium. To induce cholinergic differentiation, 10 µM retinoic acid was added to the culture medium containing 5% FBS on day 0 and maintained for five days. The culture medium was refreshed on day 3. Cells were used between passages 30 and 32.

After the 5-day differentiation period, insulin resistance was induced by treating the cells with 1 mM STZ. Simultaneously, cells were treated with 0.3–30 µM resveratrol or its analogs. Based on our previous study [20], 10 µM was identified as an effective and non-toxic concentration in SH-SY5Y cells; therefore, this concentration was applied for most of the assays. Dose–response curves (0.3–30 µM) were additionally performed for selected endpoints (MitoTracker, JC-1, and Acridine Orange), which are particularly sensitive to mitochondrial and lysosomal changes.

For ELISA measurements, cells were seeded in 10 cm Petri dishes at a density of 2.5 × 10^6^ cells per dish. After differentiation, insulin resistance was induced by treating the cells with 1 mM STZ. Simultaneously, cells were treated with 10 µM resveratrol or its analogs. Following 72 h of STZ treatment, cells were stimulated with 10–1000 nM insulin for 1 h before being harvested using lysis buffer containing 1 mM EDTA, 0.5% Triton X-100, and 6 M urea in PBS, supplemented with phosphatase inhibitor cocktails 2 and 3.

### 4.3. Determination of ROS Generation and Mitochondrial Membrane Depolarization

Mitochondrial membrane potential and ROS generation were assessed using JC-1, DCFDA, and hydroethidine (HE) staining, respectively. Cells were incubated with 5 µM JC-1 or 2 µM DCFDA and 1 µM HE in PBS for 30 min at 37 °C. After incubation, cells were washed with PBS, and fluorescence was measured using a Varioskan LUX microplate reader (ThermoFisher Scientific). JC-1 fluorescence was detected at 485/530 nm (green) and 530/590 nm (red), DCFDA at 485/530 nm, and HE at 510/600 nm. Mitochondrial depolarization was calculated as the ratio of green to red fluorescence. Data were normalized to nuclear staining intensity using Hoechst 33342.

### 4.4. Mitochondrial Mass Measurement

Mitochondrial content was assessed using Mitotracker Green staining. Cells were incubated with 200 nM Mitotracker Green in PBS for 30 min at 37 °C. After incubation, cells were washed with PBS, and fluorescence was measured using a Varioskan LUX microplate reader at an excitation/emission wavelength of 485/530 nm. Data were normalized to nuclear staining intensity using Hoechst 33342.

### 4.5. Acridine Orange Staining of the Acidic (Autophago-) Lysosomes

Acidic vacuole formation was assessed using acridine orange staining. Cells were incubated with 1 µM acridine orange for 15 min at 37 °C in the dark. After washing with PBS, fluorescence was visualized using an EVOS M5000 epifluorescence microscope (ThermoFisher Scientific). To evaluate autophagic activity, the red/green (660/530 nm) fluorescence intensity ratio was quantified.

### 4.6. Thioflavin S Staining for Protein Aggregates

Thioflavin S staining was performed to detect protein aggregates. It is important to note that Thioflavin S binds to β-pleated sheet-rich, amyloid-like structures in a protein-independent manner; therefore, in our SH-SY5Y model, we interpreted the signal as an indicator of global amyloid-like aggregate burden, without attributing it to a specific protein type. After treatment, cells were fixed with 4% paraformaldehyde for 1 h at room temperature, washed with PBS, and incubated with 0.1% thioflavin S solution in the dark for 10 min. Excess dye was removed with ethanol washes, and fluorescence was visualized using an EVOS M5000 epifluorescence microscope with a FITC filter set. Microscopic images were analyzed with Fiji Image v2 software (National Institute of Health, Bethesda, MD, USA), applying thresholding to exclude background signal. The fluorescence intensity ratio of thioflavin S to DAPI was calculated.

### 4.7. LDH Release Viability Assay

Cell viability was assessed by measuring LDH release using the CytoTox-ONE Homogeneous Membrane Integrity Assay (Promega, Madison, WI, USA) according to the manufacturer’s instructions. After treatment, cell culture supernatants were collected, and LDH release was determined by fluorescence measurement at 530/590 nm. Data were normalized to total LDH activity measured after complete cell lysis.

### 4.8. ELISA Measurement

Phospho-GSK-3 alpha/beta (S21/S9) levels were measured using the DuoSet IC ELISA kit (Bio-Techne, Minneapolis, MN, USA) according to the manufacturer’s protocol. Cells were lysed in a lysis buffer containing 1 mM EDTA, 0.5% Triton X-100, and 6 M urea in PBS, supplemented with phosphatase inhibitor cocktails 2 and 3. Protein content of the extract was measured by the Bradford method, and equal amounts of protein were loaded into the ELISA.

### 4.9. Statistical Analysis

All data were analyzed using GraphPad Prism 8 software (La Jolla, CA, USA). Concentration-response curves were fitted using nonlinear regression analysis. One-way ANOVA with Dunnett’s post hoc test was used for comparisons. Results are expressed as mean ± standard deviation (SD), with statistical significance set at *p* < 0.05.

## 5. Conclusions

Our findings clearly demonstrate the neuroprotective effect of the examined resveratrol analogs in an in vitro AD model based on STZ-induced damage and insulin resistance. All the compounds prevented the accumulation of protein aggregates and restored the response to insulin, which is consistent with ameliorating AD-like pathology. Resveratrol and monomethyl resveratrol had rather similar effects, while trimethyl resveratrol, which lacks direct antioxidant activity, was less potent in protecting neuron-like cells. These results suggest that the preservation of some phenolic hydroxyl groups on the stilbene core is critical for achieving optimal neuroprotective activity. Oxyresveratrol, on the other hand, showed only antioxidant activity among the studied mechanisms that may contribute to its considerable neuroprotective effect.

Our results indicate the importance of structure–activity relationship experiments in exploring the neuroprotective effect of stilbenes, as small structural differences had a significant impact on their biological activity.

## Figures and Tables

**Table 1:** Summary of the effects of resveratrol and its derivatives.

	RES	MONO	TRI	OXY
Peroxide				
at 6 h	↓	↓	−	↓
at 72 h	−	↑	−	−
Superoxide				
at 6 h	↓	−	−	↓
at 72 h	−	−	−	−
Mitochondrium membrane depolarization				
at 6 h	−	−	−	−
at 72 h	↑	↑	↑	−
Mitochondrial mass	−	−	−	−
Autophagy	↑	↑	↑	−
Protein aggregates	↓	↓	↓	↓
LDH release	↓	↓	↓	−
GSK3β phosphorylation	↓	↓	↓	↓

↑: increased; ↓: decreased; −: unaltered; RES: resveratrol; OXY: oxyresveratrol; MONO: monomethyl resveratrol; TRI: trimethyl resveratrol.

## Data Availability

The original contributions presented in this study are included in the article. Further inquiries can be directed to the corresponding author.

## Abbreviations

The following abbreviations are used in this manuscript:

AD	Alzheimer’s disease
Aβ	β-amyloid
AO	Acridine Orange
APP	Amyloid precursor protein
DAPI	4′,6-diamidino-2-phenylindole
DCFDA	2′,7′-Dichlorofluorescein diacetate
DMSO	Dimethyl sulfoxide
EC50	Half maximal effective concentration
ELISA	Enzyme-linked immunosorbent assay
FBS	Fetal bovine serum
GLUT	Glucose transporter
GSK-3β	Glycogen synthase kinase-3 beta
HE	Hydroethidine
IC50	Half maximal inhibitory concentration
IRS	Insulin receptor substrate
JC-1	5,5′,6,6′-Tetrachloro-1,1′,3,3′-tetraethylbenzimidazolylcarbocyanine iodide
LDH	Lactate dehydrogenase
LS	Low serum
MAPK	Mitogen-activated protein kinase
mTOR	Mammalian target of rapamycin
MONO	Monomethyl-resveratrol
Nrf2	Nuclear factor erythroid 2–related factor 2
OXY	Oxyresveratrol
PBS	Phosphate-buffered saline
PI3K/Akt	Phosphoinositide 3-kinase/Protein kinase B
RES	Resveratrol
ROS	Reactive oxygen species
RNS	Reactive nitrogen species
SD	Standard deviation
SIRT1	Sirtuin 1
STZ	Streptozotocin
T2DM	Type 2 diabetes mellitus
TRI	Trimethyl resveratrol
